# Nickel and cobalt: Underestimated contact allergens in hairdressers? 

**DOI:** 10.5414/ALXDB413E

**Published:** 2022-03-04

**Authors:** Cara Symanzik, Christoph Skudlik, Swen Malte John

**Affiliations:** 1 Institute for Interdisciplinary Dermatological Prevention and Rehabilitation (iDerm) at the University of Osnabrück, Osnabrück, Germany,; 2 Department of Dermatology, Environmental Medicine and Health Theory, University of Osnabrück, Osnabrück, Germany

**Keywords:** nickel, cobalt, hairdresser, metal tools, hairdressing tools, contact allergy, contact dermatitis, nickel spot test, cobalt spot test

## Abstract

Introduction: Nickel and cobalt were not regarded as pertinent contact allergens in the hairdressing trade for the last decades. It was even stated that the relevance of nickel allergy in the hairdressing trade has been overestimated for several years. Recently, nickel and cobalt release from a multitude of metal tools in the German hairdressing trade was documented in two field studies. Methods: Review of two field studies. Results: In 2019, nickel release from 9.2% of 229 tested metallic hairdressing tools was evidenced, and in 2021, nickel release from 27.6% as well as cobalt release from 2.1% of 475 tested tools was detected in overall 30 North German hairdressing salons. Tweezers, sectioning clips, hair clips, and straight razors were identified as nickel as well as cobalt releasing tools. Crochet hooks and tail combs were identified as only nickel releasing tools. Discussion: A variety of metallic tools – which are used daily by hairdressers – release nickel and/or cobalt in allergologically relevant amounts. This circumstance has to be considered problematic with regard to the development of work-related allergic contact dermatitis. Thus, nickel and cobalt should possibly receive greater attention as potential contact allergens in the hairdressing trade. Conclusion: The proven nickel and cobalt release from metallic hairdressing tools might entail legal ramifications in terms of insurance law. In case of nickel and cobalt allergies within the occupational group of hairdressers, metal tools might be considered as feasible sources for nickel and cobalt exposure.

**German version published in Dermatologie in Beruf und Umwelt, Jahrgang 69, Nr. 4/2021, S. 190-196.**

## Introduction 

Nickel and cobalt are among the most common contact allergens in the private environment [1]. In recent decades, nickel and cobalt were not considered epidemiologically relevant as potential occupational contact allergens for hairdressers. Stenveld reports that the significance of nickel allergies in the hairdressing trade was overestimated in the 1970s as well as in the 1980s [2]. Moreover, in the 1990s, it was explicitly stated that there was no increased exposure to nickel in the occupational environment among hairdressers [3]. In recent field studies, release of nickel and cobalt in allergologically relevant amounts from metallic working tools in the German hairdressing trade was proven [4, 5]. In the context of the present study, the two current field studies on nickel and cobalt release from metal tools in the German hairdressing trade will be summarized, analyzed, and evaluated. In doing so, possible insurance law implications that could result from the new scientific findings will be considered. 

## Materials and methods 

Two field studies from 2019 and 2021 were summarized, analyzed, and evaluated with regard to the applied methods as well as the generated results concerning nickel and cobalt release from metal tools in overall 30 hairdressing salons in Northern Germany. 

## Results 

Recent nickel and cobalt spot testing took place in the North German hairdressing trade [4, 5]. In 2019, metallic working tools (scissors, sectioning clips, hair rollers, tail combs, whisks, tweezers, and hand showers) were tested for nickel release by means of a so-called nickel detection test (nickel spot test; Chemo Nickel Test, Chemotechnique Diagnostics, Vellinge, Sweden) in two northern German states (Lower Saxony and North Rhine-Westphalia) [4]. In 2021, metal tools (scissors, sectioning clips, hair clips, hair rollers, tail combs, tweezers, straight razors, whisks, hand showers, and crochet hooks) were tested for nickel release with a nickel spot test (Chemo Nickel Test, Chemotechnique Diagnostics, Vellinge, Sweden) and with a so-called cobalt spot test; Chemo Cobalt Test, Chemotechnique Diagnostics, Vellinge, Sweden) for cobalt release, with testing taking place in three northern German states (Lower Saxony, North Rhine-Westphalia, and Schleswig-Holstein) [5]. In both studies, hairdressing salons in the low, medium and high price segments were included in equal numbers in the investigations. The testing areas of the respective metal tools were determined in advance in both studies to ensure standardized testing. The testing areas were selected according to the definition of the European Chemicals Agency (ECHA) for prolonged and direct skin contact [6]. An overview of central features of the methodological approaches of the aforementioned studies can be found in Table 1. 

In the study from 2019, nickel release was detected in 9.2% of the metal tools tested in total (n = 229); cobalt spot testing was not performed [4]. In 2021, nickel release was found in 27.6% and cobalt release was found in 2.1% of the metal tools tested in total (n = 475) [5]. The results of the aforementioned studies concerning nickel and/or cobalt release from metallic hairdressing tools are summarized in Table 2. 

## Discussion 

Nickel release from some metal tools used in the hairdressing trade – scissors and crochet hooks – has already been investigated in the Danish hairdressing trade [7]. In 2009, Thyssen and coworkers detected nickel release in 3.7% of 213 tools tested in the hairdressing trade: positive nickel test results were present in 1 of 200 scissors tested (0.5%) and in 7 of 13 crochet hooks tested (53.8%) [7]. Although a large number of metal tools used in the hairdressing trade were not considered in the investigation by Thyssen and coworkers, the results provided a reason to question whether further metal tools used in the hairdressing trade release nickel – despite the regulations in force (Regulation (EC) No. 1907/2006; REACH (Registration, Evaluation, Authorization and Restriction of Chemicals) Regulation) [8]. Taking into account recent data showing that both nickel and cobalt allergies are common among hairdressers, it is necessary to explore whether occupational sources of exposure should be considered as possible [9, 10]. To the best of our knowledge, the study from 2019 considered in the present work represents the first study in which nickel release from all common metallic work tools in the hairdressing trade was investigated [4]; the study from 2021 is the first study in which, in addition to nickel spot testing, an analysis regarding cobalt release from metal tools in the hairdressing trade was also performed [5]. 

Both recent studies show that a wide range of metallic work tools in hairdressing release nickel and/or cobalt [4, 5]. In the work from 2019, sectioning clips, tail combs, and tweezers were identified as nickel-releasing metal tools [4]. In the work from 2021, nickel release from sectioning clips, tail combs, and tweezers was also detected within a new collective of tools [5]. In addition, hair clips, crochet hooks, and straight razors – which were not tested in the previous study from 2019 – were identified as nickel-releasing tools [5]. Testing the metal tools simultaneously for cobalt release demonstrated that a proportion of the tools tested also released cobalt, with hair clips, tweezers, sectioning clips, and straight razors identified as cobalt-releasing tools [5]. Scissors, which were identified as nickel-releasing tools in the work from 2009 in Denmark [7], did not release nickel in both current analyses from Germany [4, 5]. Nickel and cobalt release from hair rollers, whisks, and hand showers was also not found within the recently tested collectives of metal tools [4, 5]. Within both studies considered, it was reported that positive nickel and/or cobalt test results were found in hairdressing salons of each price segment (low, medium, and high) and also of different geographical locations (Lower Saxony, North Rhine-Westphalia and Schleswig-Holstein) [4, 5]. This circumstance indicates that the results of the mentioned investigations could be valid for i) the entire German hairdressing trade as well as ii) possibly even for parts of the European hairdressing trade, since the metal tools tested in the described studies are distributed throughout Germany and partly also in further countries of the European Union (EU). 

In addition, the work from 2021 reported on the use of other metallic tools and equipment with metal parts in the hairdressing trade – measuring spoons for blonding powder, squeezers and so-called bobby pin bracelet. These sightings draw attention to the fact that – in addition to the metal tools investigated in the present studies – other potential nickel- and/or cobalt-releasing items are used in the hairdressing trade that may be unknown to persons unfamiliar with the subject. Future investigations should therefore include all potential nickel- and cobalt-releasing metal tools as well as equipment with metal parts, which is particularly relevant for items with which there is prolonged and direct skin contact. Metal objects such as crochet hooks and hair clips, with which there is prolonged and direct skin contact during the performance of special services, should also be considered here. Crochet hooks are mainly used in the context of hair coloring techniques, especially the creation of so-called cap highlights. In the case of hair clips, a frequent use that may involve prolonged and direct skin contact – which could be sufficient for the elicitation of allergic contact dermatitis – is in the creation of hairstyles by means of so-called wrapping techniques. An exclusive analysis of the main working tools – for example scissors – does not seem to be sufficient to identify potential sources of exposure to nickel and/or cobalt in the hairdressing trade in a comprehensive and targeted manner. 

Although the methods used in the presented work (nickel spot testing by dimethylglyoxime, cobalt spot testing by nitroso-R-salt) represent semi-quantitative methods, according to scientific knowledge, an allergologically relevant emission of nickel and/or cobalt ions – with regard to sensitization as well as elicitation – can be detected with these testing methods [11, 12, 13, 14]. The released nickel and/or cobalt ions are to be regarded as problematic especially in the hairdressing trade. This is due to the high level of skin strain in the hairdressing trade caused by regular wet work and frequent contact with chemicals/detergents, which results in disruption or damage to the epidermal skin barrier function, accompanied by the development of a proinflammatory skin milieu [15, 16]. This favors the genesis of allergic contact dermatitis, which is to be regarded as critical especially in the case of prolonged and direct skin contact with nickel- and cobalt-releasing objects. All nickel- and/or cobalt-releasing metal tools in the presented studies are regularly used in the daily working life of the hairdressers; their use thus potentially corresponds to prolonged and direct skin contact when performing various hairdressing services and activities [6]. Due to the fact that nickel acts as an adjuvant in the process of cobalt sensitization, a so-called co-exposure – which may be given by various metal tools identified as nickel- and cobalt-releasing at the same time – should strictly be avoided in particular [17, 18]. As no effective causal therapy is available for the treatment of contact allergy, the implementation of adequate allergen avoidance [19] should not only be considered as an effective intervention measure for already sensitized individuals; it can also be used as a preventive effort to obviate sensitization in non-sensitized individuals – especially in hairdressers, who are at a particularly high risk of developing – occupationally induced – allergic contact dermatitis. The results of the studies [4, 5] considered in the present work concerning potential sources of exposure to nickel and cobalt in the German hairdressing trade can be applied for the evidence-based design of health education measures (health education programs) as well as in the context of individual dermatological consultations. 

## Conclusions 

In the course of of two recently conducted field studies, a nickel and cobalt release from a wide range of metallic working tools in the hairdressing trade in northern Germany could be proven [4, 5]. Accordingly, the earlier assumption that nickel and cobalt are basically not relevant contact allergens in the hairdressing trade has to be critically considered and questioned. The field studies analyzed in the present work show that nickel and cobalt are present in relevant amounts in tools that are part of the basic equipment in the hairdressing trade. Whether these amounts are sufficient to cause sensitization in previously non-sensitized individuals is unclear and must be checked on a case-by-case basis; on the other hand, the probability that exposure is sufficient to cause elicitation is high. The study results should not lead to the conclusion that every nickel or cobalt sensitization in hairdressers should be regarded as occupationally acquired, which would mean that it would be included in the estimation of the amount of the reduction in earning capacity in the case of an occupational disease. Hairdressers, however, often suffer from nickel and/or cobalt allergies due to their private (non-occupational or pre-occupational) exposure. Culturally and habitually determined factors for the more frequent occurrence of nickel as well as cobalt allergies – such as ear piercing in childhood or wearing fashion jewelry potentially containing nickel and cobalt, etc. – in the female population are generally known and undisputed [20]. Nevertheless, the results of recent studies [4, 5] indicate that occupational skin contact with nickel- and/or cobalt-releasing metal objects in the hairdressing trade may also be present. These proven exposures signify an increased risk for hairdressers – and especially for already sensitized persons – with regard to the development of occupational allergic contact dermatitis, which may result in personal health effects on the one hand, as well as in high medical costs for a possibly necessary treatment and a possibly required change of profession or in the worst case even in a withdrawal from the working life on the other hand [21, 22]. 

In the event that a nickel and/or cobalt allergy is detected in hairdressers, those providing treatment (dermatologists, occupational health physicians) can arrange for the metal tools used in the workplace to be tested for nickel and/or cobalt; the prevention service of the accident insurance is a suitable point of contact. Testing of metallic work tools as well as non-metallic tools with metal parts is recommended, especially if there is prolonged and direct skin contact with them [5]. The test methods used in the presented work are to be regarded as cost-effective as well as rapid methods for the detection of allergologically relevant nickel and cobalt release. As an immediate primary preventive action recommendation, the use of alternative products – such as tweezers coated with plastic materials or tail combs made of plastic materials – may also be advisable. The data collected should give manufacturers reason to change their production processes so that metal tools do not release nickel or cobalt and plastic materials are used at least where there is direct skin contact. In the event that the use of alternative products is not feasible, reference should be made to the wearing of adequate protective gloves when handling metallic work tools [4]. 

Since the field studies analyzed in this paper have shown that there are currently metal tools on the market which do not comply with the requirements of the REACH Regulation [8], and manufacturers’ efforts to change this situation are currently not visible, the clarification of occupational exposure is currently in the foreground with regard to the insurance law implications for the German Social Accident Insurance (DGUV). This can only be done accurately if all potentially allergen-releasing objects are examined. The localization of an acute allergic contact dermatitis must be considered as a central point. After identification or rather detection of the sources of exposure, adequate prevention efforts should be initiated, which can prevent personal suffering as well as avoidable costs for the community of solidarity. The results of the studies presented in this paper [4, 5] provide important insights into potential sources of exposure to nickel and cobalt in the hairdressing trade and can be used to implement an effective and consistent allergen avoidance program. Furthermore, the results mentioned above can be used to appropriately design further prevention measures as well as health education programs with regard to the prevention of allergic contact dermatitis caused by nickel and/or cobalt among hairdressers. In addition, an allergological and occupational dermatological relevance of the results of the described field studies is also given due to the fact that nickel sensitization and cobalt sensitization is usually classified as non-occupational but almost never occupationally relevant [23, 24]. 

## Conflicts of interest 

No conflicts of interest are disclosed. 

## Funding 

None. 

**Table 1.** Summary of the key features of the methodological approaches in the studies considered [4, 5].

**Feature of the methodological approach**	**Symanzik et al. 2019 [** 4 **]**	**Symanzik et al. 2021 [** 5 **]**
Country	Germany	Germany
State	Lower Saxony and North Rhine-Westphalia	Lower Saxony, North Rhine-Westphalia, and Schleswig-Holstein
Number of hair salons visited	12	18
Price segment of the visited hairdressing salons	Low, medium, and high	Low, medium, and high
Reagent for nickel spot testing	Dimethylglyoxime (CAS 95-45-4)	Dimethylglyoxime (CAS 95-45-4)
Test solution for nickel spot testing	Chemo Nickel Test, Chemotechnique Diagnostics, Vellinge, Sweden	Chemo Nickel Test, Chemotechnique Diagnostics, Vellinge, Sweden
Reagent for cobalt spot testing	N/T	Nitroso-R salt (disodium;3-hydroxy-4-nitrosonaphthalene-2,7-disulfonate) (CAS 525-05-03).
Test solution for cobalt spot testing	N/T	Chemo Cobalt Test, Chemotechnique Diagnostics, Vellinge, Sweden
Standardization of the test areas	Yes	Yes
Number of tools tested in total	229	475
Tested tools	Scissors, sectioning clips, hair rollers, tail combs, whisks, tweezers, and hand showers	Scissors, sectioning clips, hair clips, hair rollers, tail combs, tweezers, straight razors, whisks, hand showers, and crochet hooks

N/T = not tested.

**Table 2.** Results of the currently conducted field studies regarding nickel and cobalt release from metallic working tools in the German hairdressing trade [4, 5].

	**Symanzik et al. 2019 [** 4 **]**		**Symanzik et al. 2021 [** 5 **]**			
	**Nickel positive**		**Nickel positive**		**Cobalt positive**	
Metal tools	%	n/total	%	n/total	%	n/total
Scissors	0	0/62	0	0/116	0	0/116
Hair rollers	0	0/45	0	0/60	0	0/60
Whisks	0	0/20	0	0/22	0	0/22
Hand showers	0	0/13	0	0/20	0	0/20
Tail combs	7.4	2/27	8.9	4/45	0	0/45
Straight razors	N/T	N/T	15.6	5/32	3.1	1/32
Sectioning clips	17.8	8/45	44.0	33/75	2.7	2/75
Tweezers	64.7	11/17	60.0	24/40	7.5	3/40
Crochet hooks	N/T	N/T	100	5/5	0	0/5
Hair clips	N/T	N/T	100	60/60	6.7	4/60
Total	9.2	21/229	27.6	131/475	2.1	10/475

N/T = not tested.
